# Extensive deep vein thrombosis as a complication of testicular cancer treated with the BEP protocol (bleomycin, etoposide and cisplatin): case report

**DOI:** 10.1590/S1516-31802006000600009

**Published:** 2006-11-01

**Authors:** Max Senna Mano, José Luiz Miranda Guimarães, Sören Franz Marian Chicata Sutmöller, André Borba Reiriz, Christian Sandor Svend Chicata Sutmöller, Angelo Di Leo

**Keywords:** Venous thrombosis, Testicular neoplasms, Chemotherapy, Thrombolytic agents, Pulmonary embolism, Trombose venosa, Câncer do testículo, Quimioterapia, Fibrinolíticos, Embolia pulmonar

## Abstract

**CONTEXT:**

There are no reports in the literature of massive deep venous thrombosis (DVT) associated with cisplatin, bleomycin and etoposide (BEP) cancer treatment.

**CASE REPORT:**

The patient was a 18-year-old adolescent with a nonseminomatous germ cell tumor of the right testicle, with the presence of pulmonary, liver, and massive retroperitoneal metastases. Following radical orchiectomy, the patient started chemotherapy according to the BEP protocol (without routine prophylaxis for DVT). On day 4 of the first cycle, massive DVT was diagnosed, extending from both popliteal veins up to the thoracic segment of the inferior vena cava. Thrombolytic therapy with streptokinase was immediately started. On day 2 of thrombolytic therapy, the patient developed acute renal failure, due to extension of the thrombosis to the renal veins. Streptokinase was continued for six days and the outcome was remarkably favorable.

## RESUMO

**CONTEXTO:**

Não há relatos na literatura de trombose venosa profunda (TVP) extensa associada ao protocolo de quimioterapia cisplatina, bleomicina e etoposite (BEP).

**RELATO DO CASO:**

O paciente era um adolescente de 18 anos com um tumor germinativo não-seminomatoso no testículo direito, com metástases pulmonares, hepáticas e retroperitoneais. Após orquiectomia radical, o paciente começou a receber quimioterapia de acordo com o protocolo BEP (sem profilaxia rotineira para TVP). No quarto dia do ciclo, TVP massiva foi diagnosticada, estendendo-se das veias poplíteas até o segmento inferior da veia cava torácica. Tratamento trombolítico foi iniciado imediatamente com estreptoquinase. No segundo dia da terapia trombolítica, o paciente desenvolveu insuficiência renal aguda, devido ao acometimento das veias renais pela trombose. Estroptoquinase foi mantida por seis dias e o paciente teve evolução surpreendentemente favorável.

## INTRODUCTION

Malignant diseases have been associated with a number of vascular phenomena that involve thromboembolism. Such events may result in considerable morbidity, impaired quality of life and, in some instances, may be life-threatening. This is a particularly relevant problem in some malignant diseases such as germ cell tumors (GCT), in which cure or long-term survival is expected for most patients.

The association between anticancer chemotherapy and thromboembolic phenomena was first reported among patients with breast cancer treated with the combination of cyclophosphamide, methotrexate, 5-fluorouracil and tamoxifen (CMF/tam) and among patients with head and neck cancer treated with cisplatin and bleomycin.^1-3^ Since then, similar events have been reported with a variety of anticancer agents and regimens.^4-14^ In GCT, local factors may also contribute towards the increased incidence of vascular complications observed in this disease.^5,15^

We report on the case of a patient with testicular GCT who developed massive deep venous thrombosis (DVT) during chemotherapy using the bleomycin, cisplatin and etoposide (BEP) regimen. This patient had a favorable outcome with the proposed treatment, namely thrombolytic therapy with streptokinase.

## CASE REPORT

**Figure 1:**
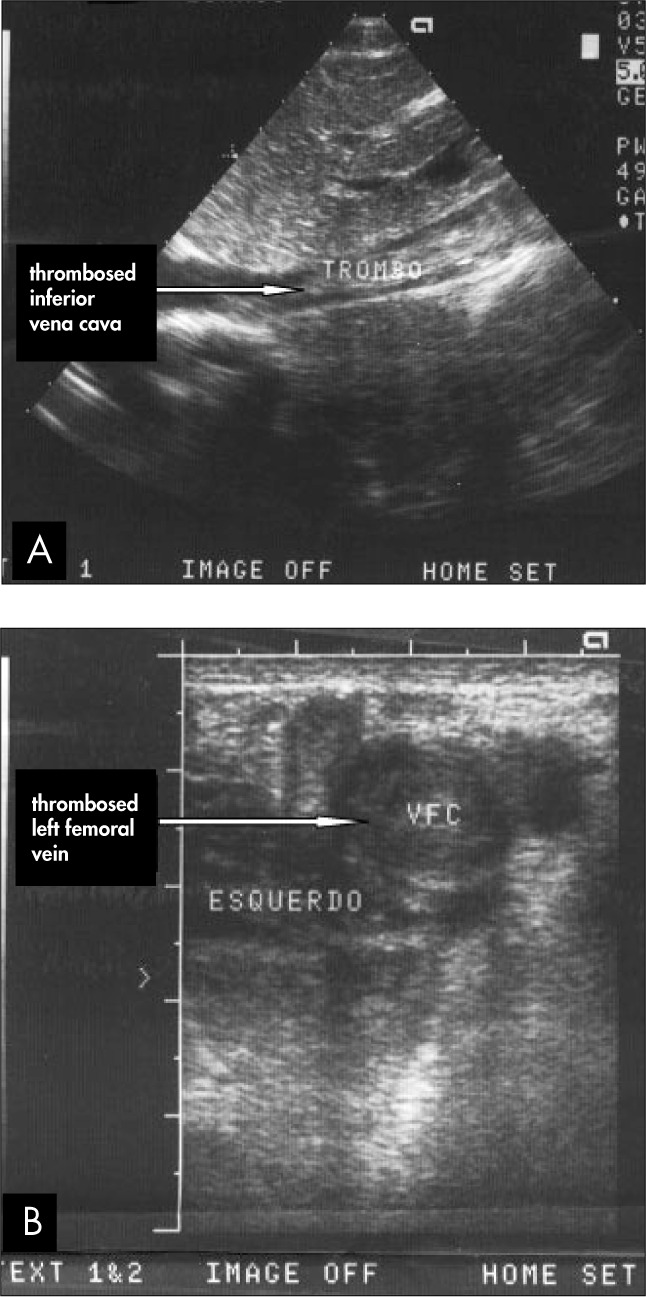
Doppler ultrasound in a 18-yearold cancer patient showing: A) massive thrombosis of the inferior vena cava; B) massive thrombosis of the left femoral vein.

The patient was 18 years old, and had been diagnosed with nonseminomatous GCT of the right testicle, of mixed histology. The computed tomography (CT) scan performed for staging revealed pulmonary and hepatic metastases, as well as massive involvement of retroperitoneal lymph nodes (causing some extrinsic compression of the inferior vena cava and renal veins). High tumor marker levels were found: lactate dehydrogenase (LDH) of 1023 mU/ml; human chorionic gonadotropin β-subunit (βhCG) of 45050 mU/ml; and alpha-fetoprotein (AFP) of 3077 ng/ml (pT3N3M1BS2, AJCC stage IIIC). Radical orchiectomy was performed and eight days postoperatively the patient started chemotherapy according to the BEP protocol (cisplatin 20 mg/m^2^ intravenously on days 1-5; bleomycin 30 UI intravenously on days 1, 8 and 15; and etoposide 100 mg/m^2^ intravenously on days 1-5). On day 4 of the first cycle, the patient developed bilateral leg pain and edema. Massive DVT was diagnosed by Doppler ultrasound (Figure 1), extending from both popliteal veins up to the thoracic segment of the inferior vena cava, close to the entrance to the right atrium. Thrombolytic therapy with streptokinase was immediately started (250,000 units intravenously over one hour as a loading dose, followed by 100,000 u/h as a continuous intravenous infusion). On day 2 of thrombolytic therapy, the patient developed acute renal failure, due to extension of the thrombosis to the renal veins (as demonstrated by Doppler echography). Hemodialysis was started, and on day 3 of thrombolytic therapy the patient showed signs of improvement. The recovery of renal function was correlated with improvement in blood flow in the renal veins and inferior vena cava. Streptokinase was stopped on day 6 of thrombolytic therapy.

The patient's symptoms, particularly pain and edema, continued to improve and follow-up Doppler echography performed on days 14 and 60 showed continued venous reperfusion. The patient continued to receive anticoagulation with warfarin (target international normalized ratio [INR] 2-3) until the end of the chemotherapy. Four cycles were given in total, which resulted in a complete clinical and biological response.

## DISCUSSION

The pathogenesis of thromboembolic phenomena in cancer patients is probably multifactorial, involving both local factors (endothelial lesions) and systemic factors (coagulation abnormalities). Oberhoff et al. demonstrated the induction of hypercoagulability by means of cyclophosphamide, methotrexate and 5-fluorouracil (CMF) in a group of patients receiving adjuvant chemotherapy for breast cancer.^16^ Similar data has been reported by other authors.^17^ Cisplatin is known to induce vasospasm, which may lead to vascular abnormalities such as Raynaud's phenomenon and systemic hypertension or, less commonly, angina, myocardial infarction, mesenteric ischemia, limb ischemia and cerebrovascular accidents.^1,5-7,9^ However, thrombosis remains the most common cause of such ischemic events. Venous thrombosis is typical, but cases of arterial thromboembolism have also been reported.^8,18-20^

The increased incidence of vascular complications observed in GCT is an intriguing finding, as these patients tend to be young and fit. Cases of secondary Raynaud's phenomenon, systemic hypertension and thromboembolism were initially reported with the PVB regimen (cisplatin, vinblastine and bleomycin),^4,6,8-10^ and similar events have also been associated with the BEP regimen.^1,20,21^ Increased long-term incidence of cardiovascular complications has also been reported in a group of cisplatin-treated GCT patients. The suggested causes were se condary metabolic and hormonal changes, such as hypercholesterolemia, hypertriglyceridemia, obesity and high levels of follicle stimulating hormone (FSH) and luteinizing hormone (LH).^22^ In a single institutional experience, the incidence of major thromboembolic complications in a group of 179 patients receiving first-line chemotherapy for GCT was as high as 8.4%. Of these, 16.5% were arterial events and 83.3% DVT, with 11 cases of pulmonary thromboembolism and one death. The same authors also suggested that high-dose corti costeroids, presence of liver metastases and an tiemetic therapy were potential risk factors.^23^

Nevertheless, a number of other predisposing factors may be present in cancer patients. In testicular cancer, for instance, local factors such as retroperitoneal lymph node metastases may cause vascular compression and stasis.^15,24^ Vascular invasion may result in endothelial damage.^25^ Even βhCG, which is a GCT marker, has already been suggested as another systemic factor predisposing to thrombosis.^4^ Recent surgery may sometimes be an additional risk factor. Although at least some of these factors may have contributed towards the development of DVT in our case, there was a clear temporal relationship between the occurrence of the event and the administration of chemotherapy.

In the setting of extensive DVT, early institution of appropriate antithrombotic therapy may prevent complications such as pulmonary thromboembolism and post-thrombotic syndrome.^26^ In such cases, heparin remains the standard treatment, although there is mounting evidence in the literature in favor of thrombolytic therapy, followed by anticoagulation.^12,27-34^ There have also been reports of patients with DVT who were successfully treated with tissue plasminogen activator as an alternative to streptokinase, although most of the time this agent has been infused locoregionally (catheter-directed) instead of systemically.^35^ The placement of a vena cava filter above the level of the thrombosis may occasionally be considered, although in view of the high complication rates observed with most of these devices, their use has most often been reserved for patients experiencing recurrent pulmonary thromboembolism despite optimal anticoagulation.^36^ In selected cases, novel surgical techniques such as percutaneous thrombectomy may be life-saving.^37^ In the case of our patient, excellent results were obtained through the administration of thrombolytic therapy with streptokinase, given as an attack dose and followed by continuous, prolonged infusion.

It is worth noting that, in a recent American consensus on the optimal use of anti-thrombotic therapy, the panel supported the use of full doses of low molecular weight heparin (LMWH) or unfractionated heparin as the initial treatment (Grade 1A). The panel also recommended against the routine use of intravenous thrombolytic treatment (Grade 1A), catheter-directed thrombolysis (Grade 1C) or venous thrombectomy (Grade 1C), but also acknowledged that selected patients (such as those with massive ileofemoral DVT and at risk of limb gangrene secondary to venous occlusion) could potentially benefit from the use of intravenous thrombolysis (Grade 2C), catheter-directed thrombolysis (Grade 1C) or venous thrombectomy (Grade 1C).^38^ Cases of massive DVT, such as the present case, were not specifically addressed by the panel, but it may be assumed they should be managed similarly to patients requiring limb-saving procedures. In view of the extent of the thrombosis, our patient was not suitable for locoregional approaches such as catheter-directed thrombolysis or venous thrombectomy.

In terms of the optimal duration of antithrombotic treatment, the current practice is to keep patients on full doses of LMWH until the end of the chemotherapy. Thereafter, decisions have to be taken on an individual basis. The American panel specifically recommended that cancer patients be kept on anticoagulation with LMWH for an additional 3-6 months after an episode of DVT.^38^ Patients who achieve cancer remission can be considered for discontinuation, while most others should remain on anticoagulation indefinitely.

## CONCLUSION

Many factors may contribute towards the development of DVT in patients with GCT. Cisplatin-based chemotherapy itself should be viewed as a highly effective but potentially thrombogenic treatment. We would point out that simple preventive measures such as prophylactic heparin may be life-saving and should be routinely considered for these patients.
